# *Clostridium difficile* Toxin B Causes Epithelial Cell Necrosis through an Autoprocessing-Independent Mechanism

**DOI:** 10.1371/journal.ppat.1003072

**Published:** 2012-12-06

**Authors:** Nicole M. Chumbler, Melissa A. Farrow, Lynne A. Lapierre, Jeffrey L. Franklin, David Haslam, James R. Goldenring, D. Borden Lacy

**Affiliations:** 1 Chemical and Physical Biology Program, Vanderbilt University School of Medicine, Nashville, Tennessee, United States of America; 2 Department of Pathology, Microbiology and Immunology, Vanderbilt University School of Medicine, Nashville, Tennessee, United States of America; 3 Department of Surgery, Vanderbilt University School of Medicine, Nashville, Tennessee, United States of America; 4 Department of Medicine, Vanderbilt University School of Medicine, Nashville, Tennessee, United States of America; 5 Department of Cell and Developmental Biology and the Epithelial Biology Center, Vanderbilt University School of Medicine, Nashville, Tennessee, United States of America; 6 Department of Pediatrics, Washington University School of Medicine, St. Louis, Missouri, United States of America; University of Illinois, United States of America

## Abstract

*Clostridium difficile* is the most common cause of antibiotic-associated nosocomial infection in the United States. *C. difficile* secretes two homologous toxins, TcdA and TcdB, which are responsible for the symptoms of *C. difficile* associated disease. The mechanism of toxin action includes an autoprocessing event where a cysteine protease domain (CPD) releases a glucosyltransferase domain (GTD) into the cytosol. The GTD acts to modify and inactivate Rho-family GTPases. The presumed importance of autoprocessing in toxicity, and the apparent specificity of the CPD active site make it, potentially, an attractive target for small molecule drug discovery. In the course of exploring this potential, we have discovered that both wild-type TcdB and TcdB mutants with impaired autoprocessing or glucosyltransferase activities are able to induce rapid, necrotic cell death in HeLa and Caco-2 epithelial cell lines. The concentrations required to induce this phenotype correlate with pathology in a porcine colonic explant model of epithelial damage. We conclude that autoprocessing and GTD release is not required for epithelial cell necrosis and that targeting the autoprocessing activity of TcdB for the development of novel therapeutics will not prevent the colonic tissue damage that occurs in *C. difficile* – associated disease.

## Author Summary

*Clostridium difficile* is an anaerobic spore-forming bacterium that infects the human colon and causes diarrhea, pseudomembranous colitis, and toxic megacolon. Most people that develop disease symptoms have undergone antibiotic treatment, which alters the normal gut flora and allows *C. difficile* to flourish. *C. difficile* secretes two toxins, TcdA and TcdB, that are responsible for the fluid secretion, inflammation, and colonic tissue damage associated with disease. The emergence of hypervirulent strains of *C. difficile* that are linked to increased morbidity and mortality highlights the need for new therapeutic strategies. One strategy is to inhibit the function of the toxins, thereby decreasing damage to the colon while the patient clears the infection with antibiotics. Toxin function is thought to depend on an autoprocessing event that releases a catalytic ‘effector’ portion of the toxin into the host cell. In the course of trying to identify small molecules that would inhibit such a function, we found that TcdB induces a rapid necrosis in epithelial cells that is not dependent on autoprocessing. The physiological relevance of this observation is confirmed in colonic explants and suggests that inhibiting TcdB autoprocessing will not prevent the colonic tissue damage observed in *C. difficile* associated diseases.

## Introduction

*Clostridium difficile* is a gram-positive, spore-forming anaerobe that infects the colon and causes a range of gastrointestinal disorders including diarrhea, pseudomembranous colitis, and toxic megacolon [1], [2]. This is a major healthcare concern as the number and severity of *C. difficile*-associated disease (CDAD) cases have increased dramatically in recent years [3]. Two large toxins, TcdA and TcdB (308 kDa and 270 kDa, respectively), are recognized as the main virulence factors of *C. difficile* [4], [5]. The C-terminal portion of these toxins is responsible for delivering an N-terminal glucosyltransferase domain (GTD) into the host cell [6], [7]. The GTD inactivates Rho family GTPases including Rho, Rac1, and Cdc42 [8], [9].

While there are numerous studies that report the effects of toxin-mediated glucosylation in cells, a consensus as to the conclusion of these reports, taken together, has been difficult due to differences in cell types, toxin concentrations, and assay methods. In addition, it appears that TcdA and TcdB can elicit different effects under similar conditions [10], [11]. In all reports, both toxins can induce a cytopathic effect characterized by cell rounding. In many reports, these cells go on to die by apoptotic mechanisms, but the time course can be up to 48 hours [12]–[19]. It has been noted, however, that apoptosis cannot be detected in cells treated with higher concentrations of TcdB [20]. In at least one study, the absence of apoptosis in cells treated with TcdB has led to suggestions of a necrotic mechanism of cell death [21].

The mechanism of GTD delivery for TcdA and TcdB involves binding a host cell receptor [22], [23], uptake by endocytosis [24], [25], pH-dependent pore formation [26]–[28], translocation across the endosomal membrane, host-factor dependent autoprocessing [29], and release of the GTD into the host cell cytosol [30]. Release is thought to allow the GTD access to the Rho-family GTPases tethered to the plasma membrane surface. An N-terminal sub-domain within the GTD is thought to serve as a membrane localization domain [31].

The autoprocessing function of the toxins is mediated by a cysteine protease domain (CPD) that follows the N-terminal GTD [32]. Inositolphosphates, predominantly inositol hexakisphosphate (InsP6), have been identified as the host factors responsible for inducing autoprocessing [29]. The InsP6-bound structures of the TcdA and TcdB CPDs reveal a positively charged InsP6-binding pocket that is distinct from the catalytic active site [33], [34]. InsP6 binding is thought to trigger conformational changes that permit the formation of the substrate-binding pocket and alignment of the catalytic residues [35]. The three catalytic amino acids Asp587, His653, and Cys698 (TcdB sequence) and the P1 substrate recognition site, Leu543, have been shown to be important for *in vitro* processing activity by genetic mutation [32]. Mutation and chemical modification of these residues has also been shown to prevent activity in various cell based assays [29], [32], [34], [36], [37]. For this reason, TcdB autoprocessing activity and GTD release have been considered important in the toxin mechanism, an idea which suggests that the CPD could serve as a useful target for novel small molecule inhibitor discovery.

The objective at the outset of this project was to conduct a high-throughput screen for small molecules that inhibit TcdB-mediated cell death. Our first step toward exploring this potential was to evaluate apoptotic and necrotic markers as cell death indicators. In observing a necrotic response to TcdB, we decided to specifically focus on the question of whether the assay would be able to detect inhibition of TcdB autoprocessing. We constructed mutant TcdB proteins with deficiencies in either the autoprocessing or glucosyltransferase activities and tested their effects on cell viability. Our unexpected observation that the mutants killed cells rapidly and at concentrations comparable to wild-type led us to investigate the role of autoprocessing and GTD release in cell death and cell rounding in greater detail. In this report, we provide evidence that epithelial cells and porcine colonic tissue challenged with TcdB undergo a rapid, necrotic cell death that is not dependent on autoprocessing and GTD release.

## Results

### TcdB induces necrosis in cultured epithelial cells

**Figure 1:**
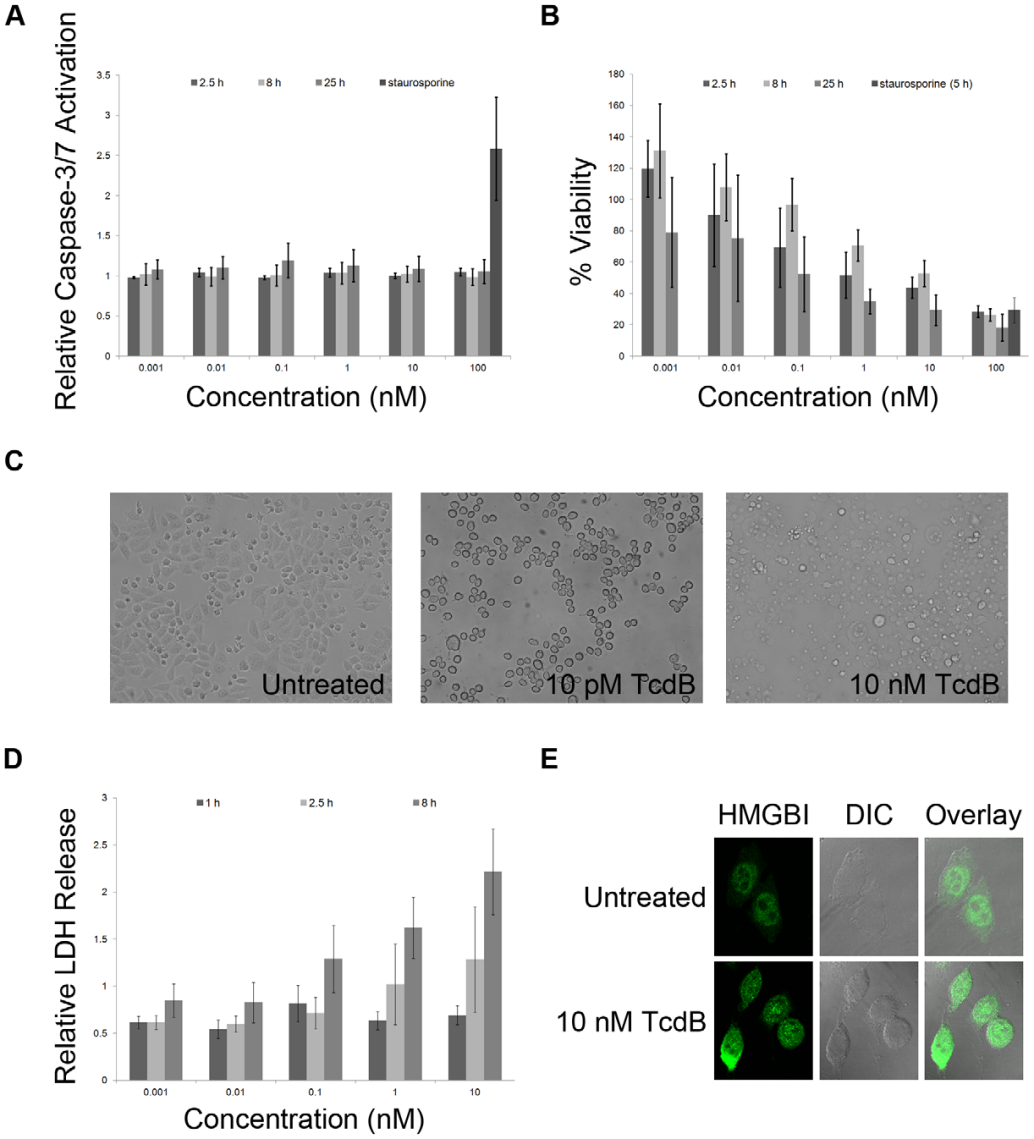
TcdB induces necrosis in epithelial cells. *A*, TcdB does not induce caspase-3/7 activation in HeLa cells, as detected by a fluorescent indicator, Apo-One. *B*, TcdB induces rapid death in HeLa cells, as detected by a luminescent indicator, CellTiterGlo. Caspase-3/7 activation and viability values represent the average of three experiments in which each condition was tested in triplicate. The error bars indicate the standard deviation between three experiments. *C*, HeLa cells were synchronized and incubated with or without TcdB for 2.5 hours at 37°C. A representative image obtained by light microscopy indicates rounding in cells treated with 10 pM TcdB and a loss of membrane integrity in cells treated with 10 nM TcdB. *D*, Extracellular LDH was detected in TcdB-treated HeLa cells after 2.5 hours using a luminescence-based indicator, Cytotox-Glo. Increased levels of LDH release were apparent after 8 hours. LDH release values represent the average of three experiments in which three replicates were averaged. Error bars indicate the standard deviation between the values obtained from the three experiments. *E*, HeLa cells were treated with a buffer control or 10 nM TcdB for 1 h and then fixed with 4% formaldehyde. Cells were stained with an antibody specific for HMGB1 and an Alexa Fluor 488 anti-mouse antibody. The cells were visualized with a LSM510 Confocal microscope. The representative images show that HMGBI is released from the nucleus of HeLa cells when treated with 10 nM TcdB and remains nuclear in the untreated cells.

The objective at the outset of this project was to conduct a high-throughput screen for small molecules that inhibit TcdB-mediated cell death. Our first goal was, therefore, to establish conditions for an assay that was sensitive and homogeneous. HeLa cells were seeded into 384 well plates and treated with TcdB at multiple concentrations for varying lengths of time. Cells were then simultaneously assayed for caspase-3/7 activation and ATP levels using fluorescent and luminescent indicators, respectively. At all concentrations and time points tested, TcdB failed to activate caspase-3 and -7, central regulators in apoptotic cell death (Figure 1A). Conversely, staurosporine, a known inducer of apoptosis, triggered significant caspase-3/7 activation at a 5 hour time point. Since the result appeared to be in conflict with a previous report showing that TcdB-treatment of HeLa cells induced an increased rate of caspase-3 activity [18], we performed additional experiments using lower toxin concentrations, a 48 hour time point, and TcdA. We did not observe caspase-3/7 activation in any of the cells treated with TcdB and only saw TcdA-induced caspase-3/7 activation when the toxin was applied at concentrations of 100 nM (Figure S1A). While our initial experiments were performed with TcdB purified from a recombinant *Bacillus megaterium* expression system, we did not observe caspase-3/7 activation when we tested TcdB purified from *C. difficile* culture supernatants (Figure S1B).

Despite the lack of caspase-3/7 activation, the TcdB treatments had a significant impact on cellular ATP levels (Figure 1B). Decreases in ATP were observed after only 2.5 hours in cells treated with 1, 10, and 100 nM TcdB suggesting that these cells were no longer viable. The effect is specific to TcdB, as TcdA only impacted the viability at concentrations of 100 nM at 24 hours (Figure S2A). While lower concentrations of TcdB can induce cell death after a 48 hour application, the effect does not appear to be dose dependent at the 48 hour time point (Figure S2A).

In an attempt to correlate the viability indicators with cytopathic events, mock and TcdB treated cells were visualized by light microscopy. At concentrations of 10 pM, a characteristic cytopathic (cell rounding) effect was observed. In contrast, cells treated with 10 nM TcdB for 2.5 hours had completely lost their membrane integrity (Figure 1C). The rapid loss of ATP and membrane integrity suggested that cells treated with nM concentrations of TcdB were dying by necrosis. To further test this hypothesis, we assessed the effect of TcdB on LDH and HMGB1 release. LDH release was apparent 2.5 hours after intoxication and at an increased level after 8 hours (Figure 1D). Similar values for LDH release are observed when the cells are treated with TcdB from *C. difficile* supernatants (Figure S2B). Notably, LDH release is only detectable at toxin concentrations above 0.1 nM, consistent with the cell death data obtained with an ATP indicator (Figure 1B). HMGB1 is a nuclear protein that is released into the cytoplasm when the cell is dying by necrosis. We found that at 10 nM TcdB, HMGB1 was released into the cytoplasm after 1 hour (Figure 1E). As a result of these studies, CellTiterGlo, the luminescent indicator of cellular ATP levels, was deemed the best indicator of cell viability for high throughput screening. The rapid loss of ATP and membrane integrity, the release of LDH and HMGB1, and the lack of caspase-3/7 activation all suggest necrosis is the mechanism of TcdB-mediated death in HeLa cells.

### Mutations in the autoprocessing domain active site and the cleavage site result in TcdB proteins with impaired autoprocessing activity *in vitro* and in cells

**Figure 2:**
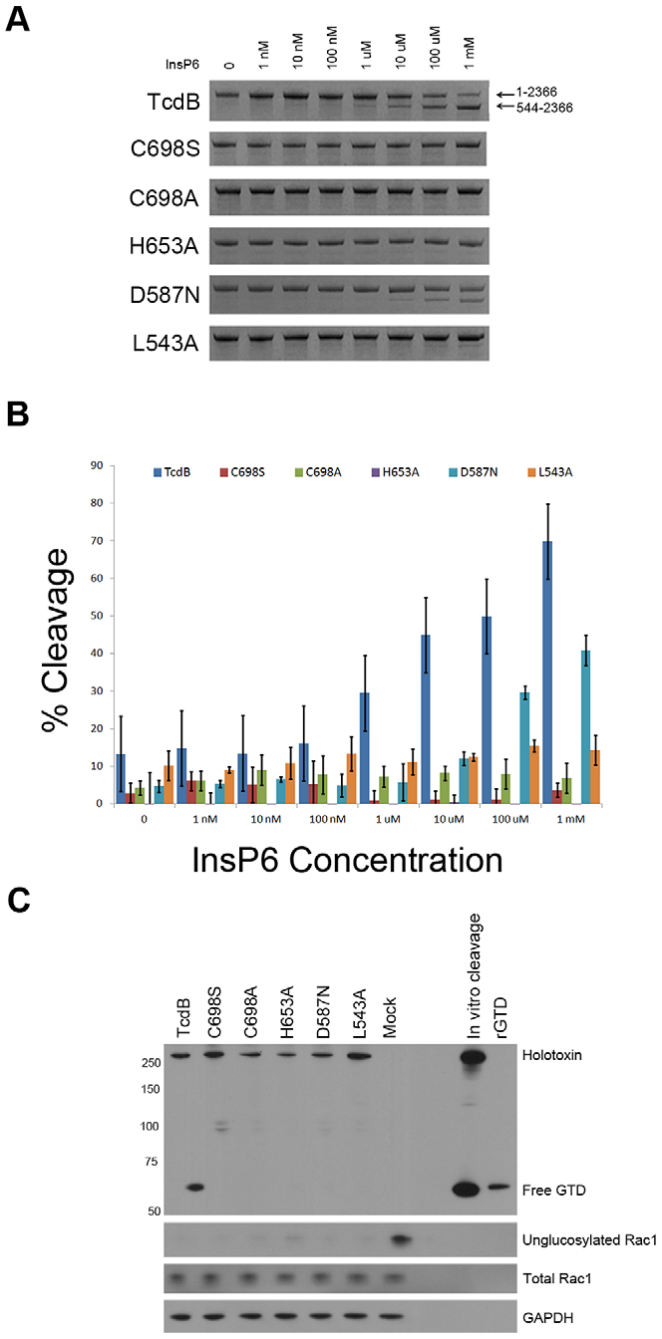
Mutations in the autoprocessing domain active site and the cleavage site result in TcdB proteins with impaired autoprocessing activity *in vitro* and in cells. *A*, Autoprocessing was induced *in vitro* by incubating wild-type TcdB and TcdB mutants with multiple InsP6 concentrations and 1 mM DTT at 37°C. After 2 hours, the proteins were subjected to SDS-PAGE and visualized with Coomassie stain. A representative series of gels is shown from experiments performed in triplicate. *B*, Three replicates of the experiments shown in panel **A** were quantified by densitometry. Bands corresponding to TcdB 544–2366 were quantified and normalized to the band corresponding to TcdB 1–2366 without InsP6. Error bars reflect the standard deviation of the percent cleavage between three experiments. The data indicate that wild-type TcdB autoproteolysis can be detected at concentrations of 1 uM to 1 mM InsP6. By comparison, TcdB mutants C698S, C698A, and H653A were completely inactive for autoprocessing at all InsP6 concentrations. The TcdB D587N and L543A had some residual activity, but autoprocessing activity was impaired relative to wild-type. *C*, GTDs of autoprocessing mutants are not released in cells. HeLa cells were synchronized for 30 minutes at 4°C, then intoxicated with 10 nM toxin. Intoxicated cells were incubated at 4°C for an hour before being moved to 37°C. Cells were harvested after 50 minutes, and cell lysates were prepared for SDS PAGE and Western blot. The blot was probed with antibodies against the TcdB GTD, unglucosylated Rac1, total Rac1, and GAPDH. While release of the GTD in cells intoxicated with wild-type TcdB was detected, the free GTD was not detected in cells treated with autoprocessing deficient mutants. The absence of signal with an antibody that recognizes unglucosylated Rac1 suggests that the autoprocessing mutants are still able to modify Rac1 in cells.

We next generated autoprocessing-deficient mutants that could be used as negative controls in a secondary assay that would allow us to select for molecules that inhibit the autoprocessing activity of the toxin. Single amino acid point mutations were made in the TcdB autoprocessing active site (C698S, C698A, H653A, and D587N) and the cleavage site (L543A). Proteins were expressed in the *B. megaterium* expression system and purified to homogeneity. All mutants were tested for their *in vitro* autoprocessing activity (Figure 2A). TcdB autoprocessing can be induced with the addition of 1 uM InsP6, and the amount of processing increases as the concentration of InsP6 increases. At all concentrations of InsP6, TcdB C698S, TcdB C698A, and TcdB H653A were completely inactive in autoprocessing, as detected by Coomassie-stained SDS PAGE (Figure 2A) and densitometry (Figure 2B). TcdB D587N and TcdB L543A had residual cleavage activity, but were significantly cleavage-impaired. Cleavage of D587N was not induced until 100 uM InsP6 was added, and the amount of processed toxin was reduced.

We next wanted to confirm that the mutants were also defective for autoprocessing in the context of the cell. HeLa cells were treated with wild-type TcdB or autoprocessing deficient TcdB mutants for 50 min, lysed, and probed by Western blot using an anti-TcdBGTD antibody. Free GTD was detected in cells treated with wild-type TcdB but was not detected in cells intoxicated with TcdB mutants (Figure 2C). The same lysates were probed with an antibody specific for unglucosylated Rac1. Rac1 is glucosylated even when the cells have been treated with autoprocessing mutants. These data suggest that in cells treated with TcdB autoprocessing mutants, the GTDs are being translocated into the cytosol, but they remain tethered to the endosome where glucosylation of Rac1 can still occur.

### Autoprocessing mutants induce necrosis in cultured epithelial cells

**Figure 3:**
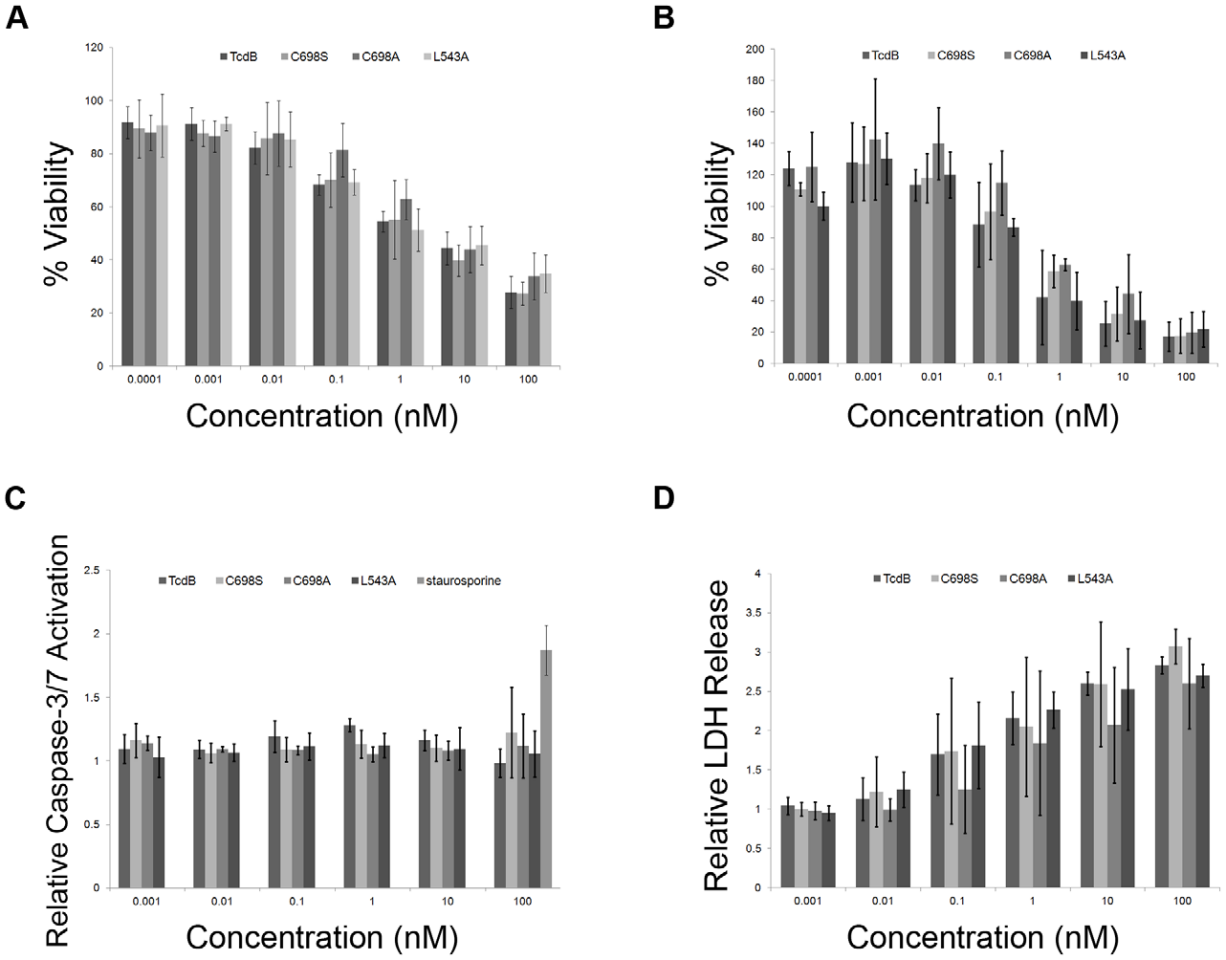
Autoprocessing mutants induce necrosis in epithelial cells. *A*, Toxins were applied to HeLa cells at concentrations ranging from 0.1 pM to 100 nM, and viability was measured after 2.5 hours with CellTiterGlo. *B*, Toxins were applied to Caco2 cells at concentrations ranging from 0.1 pM to 100 nM, and viability was measured after 18 hours with CellTiterGlo. Percent viability was determined by normalizing the signal from treated cells to the signal from untreated cells. *C*, Autoprocessing mutants did not induce caspase-3/7 activation after 25 hours. *D*, Comparable levels of extracellular LDH were detected after 8 hours in HeLa cells treated with wild-type TcdB and TcdB autoprocessing mutants. In each panel, the values represent the average of three experiments in which three replicates were averaged. Error bars indicate the standard deviation between the values obtained from the three experiments.

To test the hypothesis that small molecule inhibitors of TcdB autoprocessing could be detected in a cell based screen, we assessed cell viability in response to three of the TcdB autoprocessing mutants: TcdB C698S, TcdB C698A, and TcdB L543A. HeLa cells were treated for 2.5 hours with multiple concentrations of TcdB and the TcdB mutants, and viability was assessed using CellTiterGlo. Unexpectedly, the autoprocessing deficient mutants were found to induce cell death at concentrations comparable to TcdB (Figure 3A). To test whether this response was unique to HeLa cells, we performed similar experiments with Caco2 cells, an epithelial cell line derived from human colon. As with the HeLa cells, wild-type and autoprocessing deficient TcdB mutants induced a decrease in cellular ATP at similar concentrations in Caco2 cells (Figure 3B). Caspase-3/7 activation was not detected in HeLa cells treated for 25 hours with autoprocessing deficient TcdB mutants (Figure 3C), and the amount of LDH released in HeLa cells treated with wild-type TcdB and the TcdB C698S, C698A, and L543A autoprocessing mutants was equivalent (Figure 3D). Finally, HeLa cells were treated with 10 nM wild-type and mutant TcdB proteins in the presence of a live/dead cell indicator and imaged every 10 minutes over a 2 hour time course. A representative movie of what we observed is included in the supplemental material (Video S1). The percentage of dead cells quantified over six fields suggests that the kinetics of cell death are identical for the four proteins (Figure S3). Collectively, these data suggest autoprocessing is not required for TcdB-mediated necrosis in epithelial cells.

### TcdB induced necrosis is a glucosyltransferase independent process

**Figure 4:**
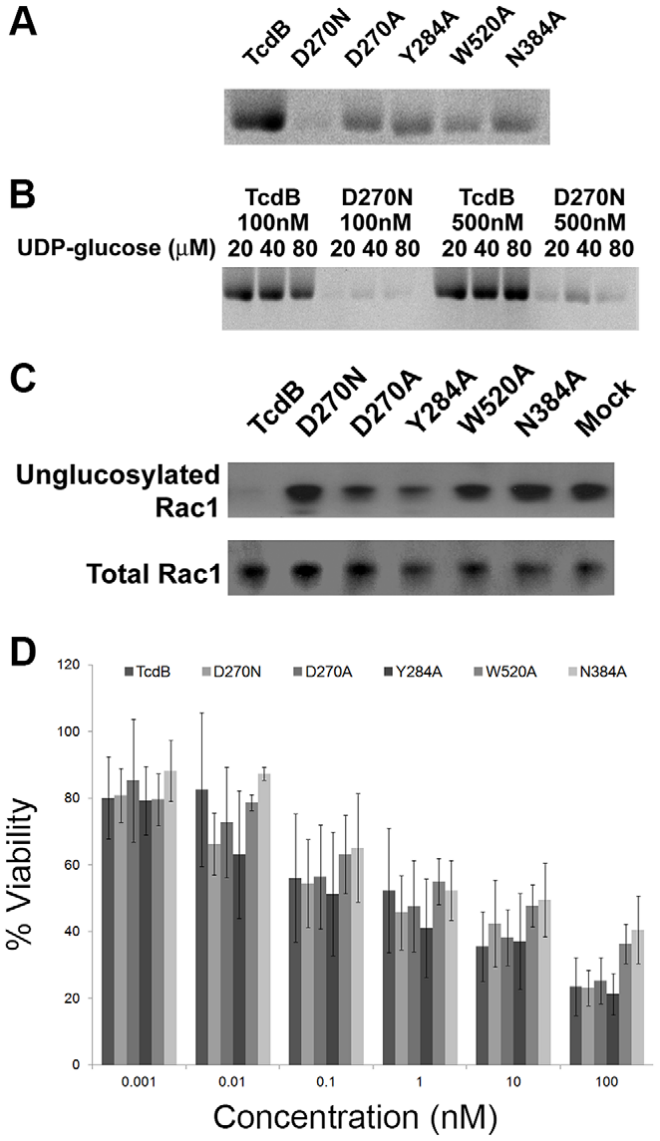
TcdB glucosyltransferase mutants cause epithelial cell death. *A*, TcdB and TcdB glucosyltransferase domain mutants (100 nM) were tested for their capacity to glucosylate purified Rac1 (2 uM) in the presence of 20 mM UDP-[^14^C]-glucose over the course of 1 h. The proteins were resolved by SDS-PAGE, and the gels were analyzed by phosphorimaging. *B*, TcdB D270N was tested with higher concentrations of both toxin and UDP-[^14^C]-glucose. Only at the highest concentrations of both toxin and UDP-[^14^C]-glucose is residual activity apparent. *C*, The TcdB glucosyltransferase mutants are impaired in their glucosyltransferase activities in HeLa cells, as determined by Western and an antibody specific for unglucosylated Rac1. *D*, Wild-type TcdB and the TcdB glucosyltransferase mutants induced comparable levels of HeLa cell death, as determined by CellTiterGlo, after 2.5 h of treatment. Percent viability was determined by normalizing the signal from treated cells to the signal from untreated cells. Values reflect the average signal from three experiments in which each condition was tested in triplicate. Error bars correspond to the standard deviation in the percent viability from the three experiments.

The idea that TcdB-induced necrosis did not require autoproteolytic release of the GTD suggested that the TcdB glucosyltransferase activity would also not be required for cytotoxicity. To test this hypothesis, single amino acid point mutations were made in the glucosyltransferase active site (D270N, D270A, Y284A, W520A, and N384A) based on the crystal structure of the TcdB GTD bound to UDP-glucose [38]. Proteins were expressed in the *B. megaterium* expression system and purified to homogeneity. All mutants were tested for their *in vitro* glucosyltransferase activity in the presence of purified Rac1 and UDP[^14^C]glucose, and all were impaired relative to wild-type (Figure 4A). Of the five mutants, the TcdB D270N mutant showed the greatest defect in *in vitro* glucosyltransfer, with residual activity only evident in the highest concentrations of enzyme and substrate (Figure 4B). Even with differences in the amount of residual activity, all five mutants were defective in the modification of Rac1 in cells (Figure 4C). Furthermore, all 5 mutants were capable of inducing a cytotoxic effect similar to that of wild-type TcdB when applied to HeLa cells (Figure 4D) and Caco-2 cells (data not shown). We interpret these data to mean that the TcdB cytotoxic effect does not require the glucosyltransferase activity of the toxin.

### The low concentration cytopathic effect is functionally distinct from the high concentration cytotoxic effect

**Figure 5:**
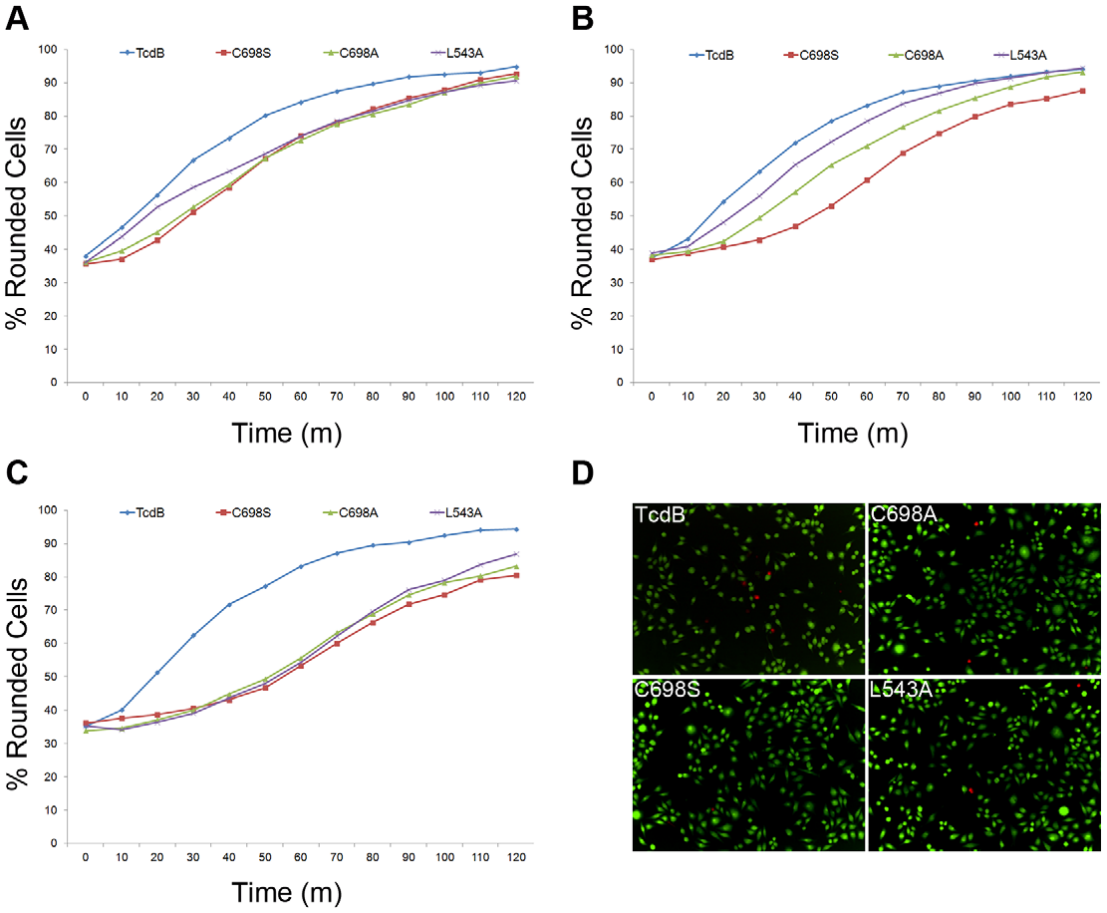
TcdB and TcdB autoprocessing mutants cause cell rounding with concentration dependent kinetics. HeLa cells were treated with multiple concentrations of wild-type and mutant TcdB proteins and imaged every 10 minutes over a 2 hour time course. The percentage of round cells was quantified over six fields for each concentration and time point. The kinetics of rounding induced by TcdB and the TcdB autoprocessing mutants is shown at concentrations of *A*, 10 pM. *B*, 100 fM and *C*, 1 fM. *D*, Representative images of cells treated with 1 fM TcdB and TcdB autoprocessing mutants for 50 minutes. Green cells are alive; red cells are dead. Images were collected with an Opera High-Throughput Confocal Screening Microscope in an environment-controlled chamber at 37°C, 5% CO_2_. Round cells were defined as having an area less than 500 um^2^ and a width-to-length ratio of less than 0.4. Analysis was performed using Columbus Analysis software.

The observation that TcdB autoprocessing mutants were able to glucosylate Rac1 in cells (Figure 2C) suggested that they would induce rearrangements in the actin cytoskeleton that result in the cytopathic ‘rounding’ phenotype. To investigate this, HeLa cells were treated with multiple concentrations of wild-type and mutant TcdB proteins and imaged every 10 minutes over a 2 hour time course. The percentage of round cells was quantified over six fields for each concentration and time point. At a 10 pM concentration, we observed similar rounding kinetics for TcdB and the three TcdB autoprocessing-deficient mutants (Figure 5A). Differences in the kinetics of rounding began to appear at a concentration of 100 fM (Figure 5B) but were not fully evident until the concentration of toxins was dropped to 1 fM (Figure 5C). The full dataset collected at concentrations spanning 8 orders of magnitude and a movie of what we observed with 10 fM wild-type TcdB is included in the supplemental material (Figure S4 and Video S2). While not required for cytotoxicity, autoprocessing and GTD release appear to be important for cytopathic processes that occur at very low concentrations. In HeLa cells, we see that at concentrations where cytopathic effects can be observed (1 fM–10 pM, Figure 5), the cells are not dead (Figure 3A). These data provide a clear distinction between the cytotoxic and cytopathic effects induced by TcdB.

### TcdB and TcdB C698A cause epithelial damage in porcine colonic explants

**Figure 6:**
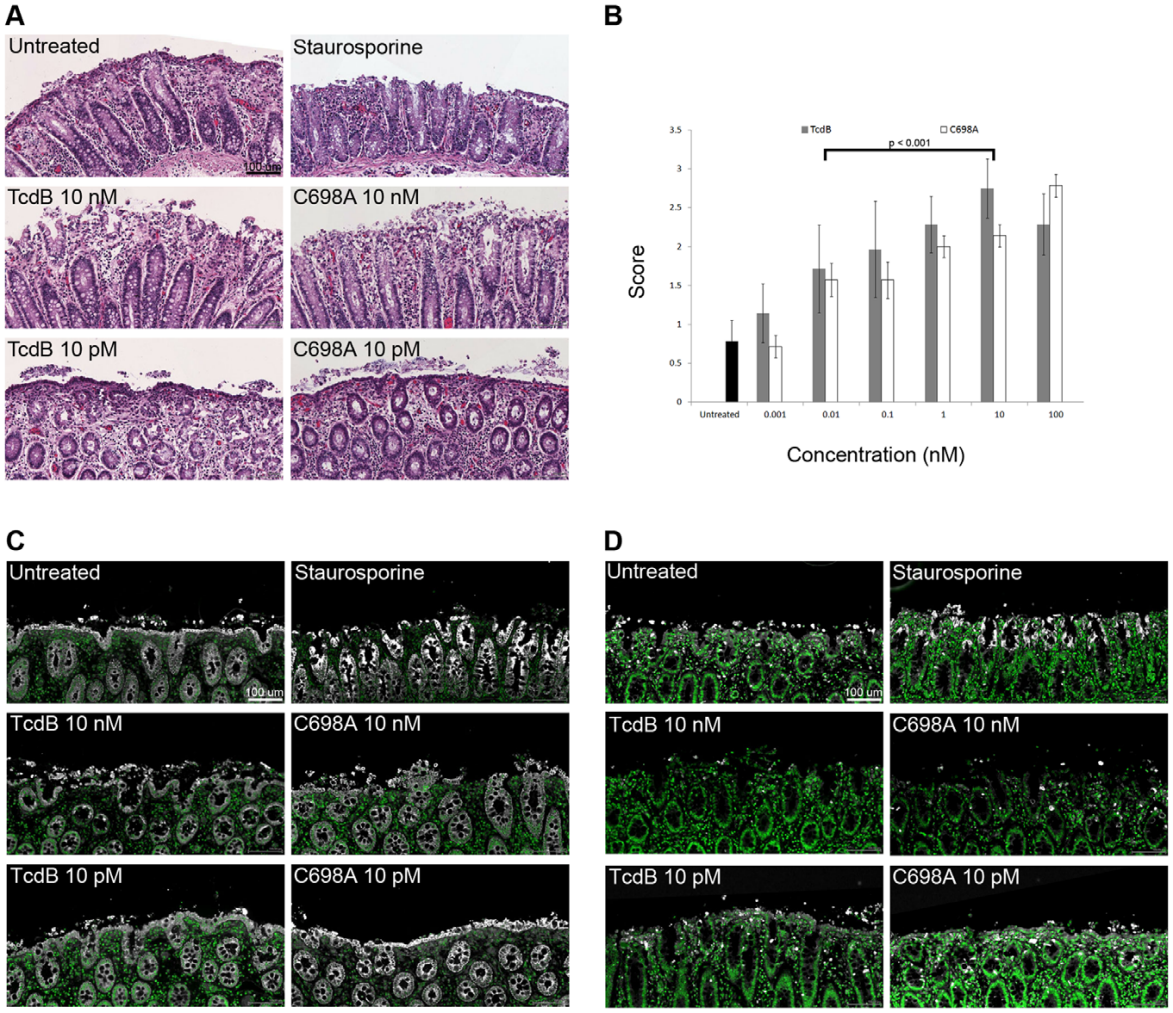
TcdB and TcdB C698A cause epithelial damage in porcine colonic explants. Porcine colonic explants were treated with 1 mM DTT to remove the mucus layer, washed with PBS, and incubated with toxin at 37°C for 5 hours. *A*, Tissue sections were stained with H&E. *B*, The H&E slides were scored in a blinded fashion using a semi-quantitative injury scale: 0- no damage; 1-superficial damage, damage limited to intact surface epithelial cells; 2-loss of up to 50% of surface epithelial cells or gland length, crypts intact; 3-loss of over 50% of surface epithelial cells and damage in greater than 50% of gland length. An injury score was calculated as the mean score for sections analyzed seven times by six individuals. Statistical analysis was performed using a two-way ANOVA and post-hoc tests. These analyses revealed a significant difference in the scores given to tissues treated with the toxins over the range of concentrations (p<0.001), while there was no statistical difference between tissues treated with TcdB and TcdB C698A. A subsequent Bonferroni's test revealed that scores given to tissue treated with 10 nM TcdB and 10 nM TcdB C698A were significantly different from scores given to tissue treated with 10 pM TcdB or 10 pM TcdB C698A (p<0.001). Error bars correspond to the standard deviation between the seven scores. *C*, The sections were also stained with an anti-pan keratin and *D*, anti-active caspase-3 antibody. Representative images of H&E, pan-cytokeratin, and active caspase-3 staining (white – pan-cytokeratin/active caspase-3, green – DAPI) show significant damage to the epithelium of the colon at concentrations of TcdB and TcdB C698A that kill cells (10 nM). At concentrations that induce rounding but not death in cultured cells (10 pM), there was no significant damage to the tissue surface cells. Caspase-3 activation was not detected at levels above background in any of the TcdB-treated tissues.

The distinction between cytopathic and cytotoxic events in cell culture led us to question if either event might correlate with disease pathology. Since the formation of necrotic lesions in the colon is a hallmark of CDAD pathology, we sought to determine the concentration of toxin required to induce these effects and whether autoprocessing was required. Porcine colonic explants were incubated with multiple concentrations of toxin for 5 hours. The tissue was fixed with formalin, embedded in paraffin, and sections were stained with H&E (Figure 6A). The slides were scored in a blinded fashion and given a score (0–3) to reflect the level of epithelial damage (Figure 6B). Damage ranged from a mostly intact surface epithelium to mucosal loss of 50% or greater in the depth of colonic crypts. The scores indicated a loss of surface epithelium in tissue treated for 5 hours with 10 nM TcdB and TcdB C698A. There was little damage in tissues treated with a buffer control or in tissues treated with wild-type TcdB and TcdB C698A at a concentration of 10 pM. Statistical analysis by two-way ANOVA revealed a significant difference in scores for tissues treated with the toxins over the range of concentrations (p<0.001), while there was no statistical difference between tissues treated with wild-type TcdB and TcdB C698A. A subsequent Bonferroni's test revealed that scores given to tissue treated with 10 nM TcdB and 10 nM TcdB C698A were significantly different from scores given to tissue treated with 10 pM TcdB and 10 pM TcdB C698A (p<0.001). The tissues were stained with an anti-pan cytokeratin antibody to confirm the keratin positive cells at the luminal surface of the colon were disrupted (Figure 6C) and an anti-activated caspase-3 antibody to confirm that the toxin treatment did not induce an apoptotic response (Figure 6D). The data reveal a correlation between the concentration of toxin required to kill epithelial cells in culture with the concentration required to disrupt epithelial integrity in colonic tissue and indicate that autoprocessing is not required for tissue damage.

## Discussion

TcdB is a multi-functional protein with a central role in CDAD pathogenesis. Our goal at the outset of this study was to conduct a screen for small molecule inhibitors that could aid in the dissection of the TcdB mechanism and the generation of new leads for therapeutic intervention. Our strategy was to combine a cell-based phenotypic screen with target-specific secondary assays. In the course of setting up our screening assays, we made two unexpected observations that warranted further investigation.

First, in contrast to a previous report [18], TcdB did not trigger the induction of apoptosis in cultured epithelial cells as measured by caspase-3/7 activation (Figure 1A, S1). Since there was an overlap in the cells, concentration of toxin, and timepoints used for analysis, we are left to speculate that the difference stems from advances in the detection reagent. The newer reagent for detecting caspase-3/7 activation allows one to directly quantitate the relative quantity of activated caspase-3/7 as opposed to the overall rate of caspase activity.

While TcdB-treatment did not induce the activation of caspase-3/7, the rapid ATP depletion observed in both HeLa (Figure 1B, 3A, S2A) and Caco2 (Figure 3B) cells suggested that the mechanism of TcdB-induced cell death was likely necrosis. The observed loss of membrane integrity (Figure 1C), rapid LDH (Figure 1D, S2B), and HMGB1 release (Figure 1E) support this conclusion.

We next questioned whether a cell-based assay for small molecule inhibitors of TcdB-induced necrosis would allow us to detect molecules that interfered with autoprocessing. We were particularly interested in targeting the autoprocessing activity of the toxin since, in theory, one could identify molecules that either activate (e.g. InsP6) or inhibit the function of the cysteine protease domain. We generated five TcdB point mutants in which key residues of the cysteine protease active site or cleavage site were mutated. Three of these mutations, C698S, C698A, and L543A, rendered TcdB non-functional for InsP6-induced autoprocessing in an *in vitro* assay, even when InsP6 was added at a 1 mM concentration (Figure 2A, 2B). The mutants were also defective for autoprocessing in the context of cells since free GTD could be detected in cells treated with wild-type TcdB but not in cells treated with the autoprocessing mutants (Figure 2C). While we cannot rule out the possibility of an alternate cleavage mechanism that results in a quantity of free GTD that is less than the detection limit of the assay, the free GTD concentration generated from such a mechanism would be too small to account for the identical cytotoxicity profiles observed in Figures 3A and 3B.

The unexpected observation that cytotoxicity does not require autoproteolytic release of the GTD led us to directly test whether the glucosyltransferase activity of the toxin was required (Figure 4). We generated five single amino acid point mutants of TcdB that differed in their residual glucosyltransferase activities *in vitro* (Figure 4A, 4B). Despite the different enzyme activity levels, all were significantly impaired relative to wild-type TcdB in their capacity to modify Rac1 in cells (Figure 4C), and all were comparable to wild-type TcdB in their cytotoxic effects (Figure 4D). These data are consistent with the observation that autoprocessing is not required and suggest that the cytotoxic response to TcdB is triggered by an event upstream of GTD release.

While not required for cytotoxicity, autoprocessing and GTD release are important for cytopathic processes that occur at low concentrations [29], [32], [34], [36], [37]. Our data are consistent with these previous reports and indicate differences in rounding kinetics emerging at concentrations of 100 fM (Figure 5C and SF4). While our Western experiment indicated TcdB autoprocessing mutants were still able to modify Rac1 in cells (Figure 2C), a similar observation has been made for a non-cleavable form of TcdA and is thought to reflect continuous vesicle trafficking and an exchange of membranous compartments that allow the uncleaved toxin to come into contact with the membrane-bound GTPases [39]. This capacity to modify Rac1 while still tethered to the endosomal membrane presumably accounts for the similar rounding kinetics that we observed when the TcdB autoprocessing mutants were applied to HeLa cells at concentrations of 1 pM and higher (Figure 5A, S4).

The concentrations of TcdB needed to induce cytopathic effects (≤1 fM, Figure S4) are significantly lower than what is required to induce the cytotoxic effect (1 nM, Figure 3). At a concentration of 10 pM TcdB, the cells are clearly round (Figure 5A) but not dead (Figure 3). The distinction between cytopathic and cytotoxic events in cell culture raises the question of whether either process correlates with mechanisms of pathology observed in the host. To address this question, we decided to test what concentration of toxin was required to induce epithelial cell damage in colonic tissue explants. Visual assessment of H&E stained colonic tissue integrity in a blinded fashion indicated damage with treatments of 10 nM TcdB but not with 10 pM TcdB (Figure 6). Similar observations were made with the TcdB C698A mutant suggesting that the damage that occurs to colonic tissue in response to TcdB does not depend on the autoprocessing activity. Pan-cytokeratin staining confirmed that the cells on the luminal surface of the tissue remained intact in the presence of 10 pM TcdB or TcdB C698A but were being disrupted in samples treated with 10 nM TcdB, 10 nM TcdB C698A, or 100 uM staurosporine. The staurosporine control revealed strong caspase-3 activation into the crypts (Figure 6D). The untreated control tissue demonstrated a low level of caspase-3 activation in the cells on the luminal surface and strong activation in single cells coming off the surface of the tissue. Tissues treated with 10 pM TcdB and TcdB C698A showed caspase-3 activation levels similar to those of the untreated tissue. Tissue treated with 10 nM TcdB or TcdB C698A demonstrated even lower levels of caspase-3 activation, presumably because the cells on the luminal surface have been shed. Unlike the untreated, staurosporine-treated, and 10 pM TcdB-treated tissues, caspase-3 activation was generally not observed in the cells that were in the process of being shed in tissues treated with 10 nM TcdB or TcdB C698A (Figure 6D). This suggests that tissue damage is not only independent of autoprocessing activity, but also not likely due to apoptosis.

The phenotypic differences with concentration led us to wonder what concentration of toxin is present in the colons of individuals experiencing the symptoms of CDAD. We found only one published report, where TcdB was quantitated using a real-time cell analysis system [40]. In this report, the TcdB concentrations in stool samples from 10 patients experiencing mild to severe symptoms of CDAD ranged from 4.9 pM to 413 pM with a mean concentration of 146 pM. Presumably, the concentration of TcdB would be much higher at the colonic epithelium prior to dilution by diarrhea. Of note, the average TcdB concentration in samples from 9 individuals who were not experiencing CDAD symptoms was 1 pM, with a range of 0.1 pM to 3.3 pM. This analysis suggests that the cytotoxic effects observed in cells and tissues treated with 1 to 10 nM TcdB are better correlated with pathology than the cytopathic effects that are induced at 1 fM concentrations.

Our data suggest that inhibiting TcdB autoprocessing will not prevent the colonic tissue damage observed in *C. difficile* associated diseases. However, while the colonic epithelium is the primary barrier separating *C. difficile* from the host, it is possible that the autoprocessing function of TcdB is important in another setting relevant to pathogenesis. For example, the colonic explant model used in this study does not account for the impact of the toxins on inflammation or the potential impact of an anaerobic environment. Evaluating the effect of autoprocessing- and glucosyltransferase-deficient toxins in an animal model of *C. difficile* infection therefore represents a priority for future studies. In addition, it will be important to define the mechanism of TcdB-mediated necrosis in cells and tissue. Relevant comparisons may come from the study of other toxins. For example, the *Bordetella pertussis* adenylate cyclase (AC) toxin is known to have multiple mechanisms that contribute to cytotoxicity [41]. Identifying the autoprocessing- and glucosyltransferase-dependent and –independent aspects of TcdB-mediated pathology represents an exciting path for future study.

## Materials and Methods

### Ethics statement

This study was performed in strict accordance with the recommendations in the Guide for the Care and Use of Laboratory Animals of the National Institutes of Health. Animal husbandry and experimental procedures related to the porcine colonic explants were performed in accordance with the Vanderbilt University Institutional Animal Care and Use Committee (IACUC) policy. Discarded colon tissues were obtained from pigs following euthanization at the end of IACUC-approved animal use protocols. Animal husbandry and experimental procedures related to the generation of the anti-TcdBGTD monoclonal antibody were performed in accordance with the Washington University Animal Studies Committee policy, approval number 20100113.

### Expression of recombinant proteins

Single amino acid point mutations were made in the TcdB autoprocessing active site (C698S, C698A, H653A, and D587N), the cleavage site (L543A), and the glucosyltransferase domain (D270N, D270A, Y284A, N384A, and W520A) using the QuickChange mutagenesis protocol (Stratagene). The template for mutagenesis and clone for the production of wild-type TcdB was a *B. megaterium* expression vector encoding the strain 10643 of TcdB [42]. A similar clone was used for expression of recombinant TcdA [42]. Plasmids for expressing TcdA, TcdB, and TcdB point mutants were transformed into *B. megaterium* according to the manufacturer's instructions (MoBiTec, Göttingen, Germany). 1 L of LB was inoculated with 35 mL overnight culture and 10 mg/L tetracycline and grown at 37°C and 230 rpm. At an OD_600_ of 0.3, expression was induced with 5 g of D-xylose. Cells were harvested after 4 h by centrifugation and resuspended in 20 mM Tris, pH 8.0, 500 mM NaCl and protease inhibitors. Cells were lysed by French press, and lysates were centrifuged at 48,000 g for 25 min. The proteins were purified by Ni-affinity chromatography, Q-sepharose anion exchange chromatography, and gel filtration chromatography in 20 mM HEPES, pH 6.9, 50 mM NaCl.

### Protein purification from *C. difficile*

Proteins were expressed and purified as previously described [1].

### Cell death assays

HeLa and Caco2 cells (cultured in DMEM, 10% FBS, 5% CO_2_ and MEM, 10% FBS, 5% CO_2_, respectively) were seeded in a black 384-well plate at a concentration of 3,000 or 1,000 cells/well, respectively. HeLa cells were intoxicated the next day, and Caco2 cells were intoxicated 36 h later. After intoxication, the cells were incubated at 37°C, 5% CO_2_ for either 2.5 h (HeLa) or 18 h (Caco2). The amount of ATP (cell viability) was assessed with a luminescence-based indicator, CellTiterGlo (Promega). LDH release was assessed with a luminescence-based indicator, CytoToxGlo (Promega). Caspase-3/7 activation was determined using a fluorescent indicator, Apo-One (Promega). Staurosporine (Sigma, 1 mM) was used as a positive control for caspase-3/7 activation. Plates were read in a Biotek Synergy 4 plate reader.

### HMGB1 release

HeLa cells were seeded into a tissue culture treated chamber slide at 2×10^4^ cells per well and incubated overnight. Cells were synchronized at 4°C and intoxicated with 10 nM TcdB for 1 h. Cells were then shifted to 37°C for 1 h. Media was removed from the cells, and the cells were washed with PBS. They were fixed with 4% paraformaldehyde at room temperature for 10 minutes and quenched with 1 mM glycine. Cells were permeated with 0.2% Triton X-100 in PBS for 5 minutes, washed in PBS, and blocked for 30 minutes in PBS, 2% BSA, 0.1% Tween 20. Cells were stained with a monoclonal antibody against HMGBI (Abcam, ab77302), and an Alexa Fluor 488 anti-mouse antibody (Invitrogen, A11001). Cells were visualized with an LSM 510 Confocal microscope.

### *In vitro* cleavage assay

1 uL InsP6 stock solution (100×) or buffer was added to 200 nM TcdB or TcdB autoprocessing mutant and incubated for 2 h at 37°C. The reactions were stopped with the addition of loading buffer and boiling and analyzed by Coomassie stained SDS PAGE.

### Anti-TcdBGTD antibody generation

Genomic DNA of *C. difficile* clinical isolate 630 was obtained from American Type Culture Collection, and the region encoding residues 1 to 549 of TcdB, which is known to encode the substrate binding and enzymatic domains of the toxin, was amplified in frame with a carboxy-terminal (His)6-tag using upstream primer:5′- CCGGATGTACAGTTGAGGGGGTAAAATGAGTTTAGTTAATAGAAAACAGTTAG -3′ and downstream primer 5′- GGTCCTCAATGATGGTGATGGTGATGAAGATTATCATCTTCACCAAGAGAACC -3′. The resulting product was cloned into plasmid pcDNA3.1 (Invitrogen, Carlsbad CA) and sequenced to ensure fidelity of the amplified product. The gene was then released with restriction enzymes BsrG1 and AgeI and cloned into similarly digested vector pHIS1525 (MoBiTec), placing the gene under control of a xylose-inducible promoter. Recombinant protein was expressed in *B. megaterium* and purified by sequential nickel affinity and gel filtration chromatography. Two mice were immunized bi-weekly by intraperitoneal injection with 100 µg purified TcdB-GTD. Three days after the third vaccination, splenocytes were harvested and fused to P3X63Ag8.6.5.3 myeloma cells using polyethylene glycol 1500 [43]. Hybridomas producing anti-TcdB-GTD MAbs were identified by ELISA, subcloned by limiting dilution, and purified by protein G immunoaffinity chromatography.

### Cell based cleavage assay

HeLa cells were synchronized by cooling to 4°C and then intoxicated with 10 nM TcdB, autoprocessing mutant, or buffer. The cells were returned to 4°C for 1 h, and then shifted to 37°C for 50 min. The cells were harvested and lysed, samples were boiled, and proteins were separated by SDS PAGE. Samples were analyzed by Western with primary antibodies specific for the TcdB GTD, unglucosylated Rac1 (BD, 610650), total Rac1 (Millipore, clone 23A8), and GAPDH (Santa Cruz Biotechnology, sc-25778). Binding of an anti-mouse, HRP-conjugated secondary antibody (Jackson ImmunoResearch Laboratories, 115-035-174) was detected with a LumiGLO kit (Cell Signaling) according to manufacturer's instructions.

### *In vitro* glucosyltransferase assay

Unless otherwise noted, 100 nM TcdB or TcdB glucosyltransferase mutants and 2 uM Rac1 were mixed with 20 mM UDP-[^14^C]glucose (250 mCi/mmol, Perkin Elmer) in a total reaction volume of 10 uL. The buffer contained 50 mM HEPES pH 7.5, 100 mM KCl, 1 mM MnCl_2_, 2 mM MgCl_2_, and 0.1 mg/mL BSA. Reactions were incubated at 37°C for 1 h and stopped with the addition of loading buffer and boiling. Proteins were separated by SDS PAGE, and glucosylation of Rac1 was detected by phosphorimaging.

### Kinetic assays of cytotoxic and cytopathic events

HeLa cells were seeded in a black 96-well imaging plate (PerkinElmer) and incubated overnight. Cells were pretreated with live/dead cell imaging dyes (Molecular Probes, R37601) and then treated with multiple concentrations of wild-type and mutant TcdB proteins. Cells were imaged in an environment-controlled chamber (37°C, 5% CO_2_) every 10 minutes over a 2 hour timecourse using an Opera High-Throughput Confocal Screening Microscope and Peltier-cooled, confocal CCD cameras. The percentage of dead cells and round cells was quantified over six fields for each concentration and time point using the Columbus Analysis software. Dead cells were defined as red cells with an intensity greater than 450 relative units, and round cells were defined as having an area less than 500 um^2^ and a width-to-length ratio of less than 0.4.

### Porcine colonic explants

Colonic tissue was harvested from purpose-bred 25–35 kg, male or female, York-Landrace crossbred pigs. Following an overnight fast and immediately after euthanasia, a midline incision was performed and 15 cm of distal colon proximal to the rectum was excised and placed in PBS. The colon was opened, the luminal side was washed 3×5 min in 1 mM DTT to remove the mucus, and 3×5 min in PBS prior to dissection. Individual tissue sections were placed in wells of a 24-well plate. A nutrient buffer [44] containing (mM/liter): 122.0 NaCl, 2.0 CaCl_2_, 1.3 MgSO_4_, 5.0 KCl, 20.0 glucose, 25.0 NaHCO_3_ (pH 7.5) was pre-conditioned with HeLa cells overnight at 37°C and used to dilute the toxins. Explants were treated with wild-type TcdB, mutant TcdB, staurosporine (100 uM, Enzo Life Sciences, ALX-380-014-C250) or nutrient buffer for 5 hours at 37°C. The tissues were fixed with formalin for 56 h, washed in PBS, and transferred to cassettes. The tissue blocks were then embedded in paraffin, and 4 µm sections were cut and stained with hematoxylin and eosin (H&E) by the Vanderbilt University Translational Pathology Shared Resource core. Stained sections were coded and evaluated by six individuals, using a semi-quantitative injury scale: 0- no damage; 1-superficial damage, damage limited to intact surface epithelial cells; 2-loss of up to 50% of surface epithelial cells or gland length, crypts intact; 3-loss of over 50% of surface epithelial cells and damage in greater than 50% of gland length. An injury score was calculated as the mean score for sections evaluated seven times by six individuals. Statistical analysis was performed using a two-way ANOVA and Bonferroni's test. For keratin and caspase staining, sections were de-paraffinized with Histo-clear (National Diagnostics) and antigens were retrieved by citric acid. The sections were blocked with Serum-free protein block (Dako), stained with a rabbit anti-pan cytokeratin or anti-active caspase-3 antibody (Santa Cruz Biotechnology, sc-15367; Abcam, ab13847), and diluted in Dako's antigen diluent with background reducing components overnight at 4°C. The sections were washed with PBS and incubated for 1 hr at RT with an AlexaFluor 546 donkey anti-rabbit antibody (Invitrogen A10040). The sections were washed with PBS and mounted with Prolong Gold with DAPI (Invitrogen). H&E, pan-cytokeratin, and caspase-3 stained sections were imaged using an Ariol SL-50 (Epithelial Biology Center Imaging Core).

## Supporting Information

Figure S1 **TcdA activates caspase-3/7 while both recombinant and native TcdB do not.** *A*, TcdB does not induce caspase-3/7 activation in HeLa cells, as detected by a fluorescent indicator, Apo-One, at 24 or 48 h. TcdA, however, does induce caspase-3/7 activation at a concentration of 100 nM at 24 h and 10 and 100 nM at 48 h. *B*, TcdB purified from *C. difficile* supernatant looks similar to TcdB purified from *B. megaterium* in that neither induce caspase-3/7 activation. Values represent the average of 3 independent experiments in which each condition was tested in triplicate. Error bars represent the standard deviation of the average of the three independent experiments.
(TIF)

Figure S2 **TcdB is more cytotoxic than TcdA, and the effects of native and recombinant TcdB on LDH release are similar.** *A*, TcdB induces significant HeLa cell death, as detected by CellTiterGlo, in 24 h at concentrations of 1, 10, and 100 nM. At 48 h, a loss of cell viability was observed at lower concentrations in a dose-independent fashion. TcdA induces significant cell death at 24 h and 48 h at a concentration of 100 nM. *B*, TcdB purified from *C. difficile* and *B. megaterium* induce release of LDH starting at 2.5 h, with increased levels apparent after 8 h of treatment. Values represent the average of 3 independent experiments in which each condition was tested in triplicate. Error bars represent the standard deviation of the three independent experiments.
(TIF)

Figure S3 **TcdB and TcdB autoprocessing mutants have the same cytotoxicity kinetics.** *A*, TcdB, TcdB C698S, TcdB C698A, and TcdB L543A at 10 nM induce HeLa cell death at similar rates, as detected by Live/Dead Cell Imaging dyes. Values represent the number of red (dead) cells per total number of cells (red+green) over six image fields and were calculated using Columbus Analysis Software. Dead cells were defined as having a red intensity greater than 450 relative units. *B*, Representative pictures of TcdB treated cells at 0 and 120 minutes. Images were taken using an Opera High-Throughput Confocal Screening Microscope.
(TIF)

Figure S4 **TcdB and TcdB autoprocessing mutants have different cytopathic kinetics at 1 fM.** HeLa cells were treated with multiple concentrations of wild-type and mutant TcdB proteins and imaged every 10 minutes over a 2 hour time course. The percentage of round cells was quantified over six fields for each concentration and time point. Percent rounded cells induced by TcdB and autoprocessing mutants is shown at concentrations of *A*, 10 nM, *B*, 1 nM, *C*, 100 pM, *D*, 10 pM, *E*, 1 pM, *F*, 100 fM, *G*, 10 fM, and *H*, 1 fM. Differences in the rounding kinetics between TcdB and autoprocessing mutants begin to appear at a concentration of 100 fM and are clearly distinct at 1 fM. Images were collected with an Opera High-Throughput Confocal Screening Microscope in an environment-controlled chamber at 37°C, 5% CO_2_. Round cells were defined as having an area less than 500 um^2^ and a width-to-length ratio greater than 0.4. Analysis was performed using Columbus Analysis software.
(TIF)

Video S1 **Cytotoxicity induced by TcdB.** HeLa cells were treated with 10 nM TcdB and imaged every 10 minutes over a 2 hour time course. The 13 frames, reported in SF3, are compiled to show how cells change with time. Green cells are alive; red cells are dead.
(MP4)

Video S2 **Cytopathy induced by TcdB.** HeLa cells were treated with 10 fM TcdB and imaged every 10 minutes over a 2 hour time course. The 13 frames, reported in SF4, are compiled to show how cells change with time. Green cells are alive; red cells are dead.
(MP4)

## References

[1] Lyerly DM, Krivan HC, Wilkins TD (1988) Clostridium difficile: its disease and toxins. Clin Microbiol Rev 1: 1–18. 3144429 10.1128/cmr.1.1.1 PMC358025

[2] McFarland LV, Stamm WE (1986) Review of Clostridium difficile-associated diseases. Am J Infect Control 14: 99–109. 3524319 10.1016/0196-6553(86)90018-0

[3] Kelly CP, LaMont JT (2008) Clostridium difficile–more difficult than ever. N Engl J Med 359: 1932–1940. 18971494 10.1056/NEJMra0707500

[4] Lyras D, O'Connor JR, Howarth PM, Sambol SP, Carter GP, et al (2009) Toxin B is essential for virulence of Clostridium difficile. Nature 458: 1176–1179. 19252482 10.1038/nature07822 PMC2679968

[5] Kuehne SA, Cartman ST, Heap JT, Kelly ML, Cockayne A, et al (2010) The role of toxin A and toxin B in Clostridium difficile infection. Nature 467: 711–713. 20844489 10.1038/nature09397

[6] Hofmann F, Busch C, Prepens U, Just I, Aktories K (1997) Localization of the glucosyltransferase activity of Clostridium difficile toxin B to the N-terminal part of the holotoxin. J Biol Chem 272: 11074–11078. 9111001 10.1074/jbc.272.17.11074

[7] Rupnik M, Pabst S, Rupnik M, von Eichel-Streiber C, Urlaub H, et al (2005) Characterization of the cleavage site and function of resulting cleavage fragments after limited proteolysis of Clostridium difficile toxin B (TcdB) by host cells. Microbiology 151: 199–208. 15632438 10.1099/mic.0.27474-0

[8] Just I, Wilm M, Selzer J, Rex G, von Eichel-Streiber C, et al (1995) The enterotoxin from Clostridium difficile (ToxA) monoglucosylates the Rho proteins. J Biol Chem 270: 13932–13936. 7775453 10.1074/jbc.270.23.13932

[9] Just I, Selzer J, Wilm M, von Eichel-Streiber C, Mann M, et al (1995) Glucosylation of Rho proteins by Clostridium difficile toxin B. Nature 375: 500–503. 7777059 10.1038/375500a0

[10] Lyerly DM, Lockwood DE, Richardson SH, Wilkins TD (1982) Biological activities of toxins A and B of Clostridium difficile. Infect Immun 35: 1147–1150. 7068215 10.1128/iai.35.3.1147-1150.1982 PMC351167

[11] Lima AA, Lyerly DM, Wilkins TD, Innes DJ, Guerrant RL (1988) Effects of Clostridium difficile toxins A and B in rabbit small and large intestine in vivo and on cultured cells in vitro. Infect Immun 56: 582–588. 3343050 10.1128/iai.56.3.582-588.1988 PMC259330

[12] Brito GA, Fujji J, Carneiro-Filho BA, Lima AA, Obrig T, et al (2002) Mechanism of Clostridium difficile toxin A-induced apoptosis in T84 cells. J Infect Dis 186: 1438–1447. 12404159 10.1086/344729

[13] Brito GA, Carneiro-Filho B, Oria RB, Destura RV, Lima AA, et al (2005) Clostridium difficile toxin A induces intestinal epithelial cell apoptosis and damage: role of Gln and Ala-Gln in toxin A effects. Dig Dis Sci 50: 1271–1278. 16047471 10.1007/s10620-005-2771-x

[14] Carneiro BA, Fujii J, Brito GA, Alcantara C, Oria RB, et al (2006) Caspase and bid involvement in Clostridium difficile toxin A-induced apoptosis and modulation of toxin A effects by glutamine and alanyl-glutamine in vivo and in vitro. Infect Immun 74: 81–87. 16368960 10.1128/IAI.74.1.81-87.2006 PMC1346681

[15] Nottrott S, Schoentaube J, Genth H, Just I, Gerhard R (2007) Clostridium difficile toxin A-induced apoptosis is p53-independent but depends on glucosylation of Rho GTPases. Apoptosis 12: 1443–1453. 17437185 10.1007/s10495-007-0074-8

[16] Gerhard R, Nottrott S, Schoentaube J, Tatge H, Olling A, et al (2008) Glucosylation of Rho GTPases by Clostridium difficile toxin A triggers apoptosis in intestinal epithelial cells. J Med Microbiol 57: 765–770. 18480335 10.1099/jmm.0.47769-0

[17] Matte I, Lane D, Cote E, Asselin AE, Fortier LC, et al (2009) Antiapoptotic proteins Bcl-2 and Bcl-XL inhibit Clostridium difficile toxin A-induced cell death in human epithelial cells. Infect Immun 77: 5400–5410. 19797069 10.1128/IAI.00485-09 PMC2786492

[18] Qa'Dan M, Ramsey M, Daniel J, Spyres LM, Safiejko-Mroczka B, et al (2002) Clostridium difficile toxin B activates dual caspase-dependent and caspase-independent apoptosis in intoxicated cells. Cell Microbiol 4: 425–434. 12102688 10.1046/j.1462-5822.2002.00201.x

[19] Matarrese P, Falzano L, Fabbri A, Gambardella L, Frank C, et al (2007) Clostridium difficile toxin B causes apoptosis in epithelial cells by thrilling mitochondria. Involvement of ATP-sensitive mitochondrial potassium channels. J Biol Chem 282: 9029–9041. 17220295 10.1074/jbc.M607614200

[20] Fiorentini C, Fabbri A, Falzano L, Fattorossi A, Matarrese P, et al (1998) Clostridium difficile toxin B induces apoptosis in intestinal cultured cells. Infect Immun 66: 2660–2665. 9596731 10.1128/iai.66.6.2660-2665.1998 PMC108253

[21] Lica M, Schulz F, Schelle I, May M, Just I, et al (2011) Difference in the biological effects of Clostridium difficile toxin B in proliferating and non-proliferating cells. Naunyn Schmiedebergs Arch Pharmacol 383: 275–283. 21212934 10.1007/s00210-010-0595-5

[22] von Eichel-Streiber C, Sauerborn M (1990) Clostridium difficile toxin A carries a C-terminal repetitive structure homologous to the carbohydrate binding region of streptococcal glycosyltransferases. Gene 96: 107–113. 2148295 10.1016/0378-1119(90)90348-u

[23] Dove CH, Wang SZ, Price SB, Phelps CJ, Lyerly DM, et al (1990) Molecular characterization of the Clostridium difficile toxin A gene. Infect Immun 58: 480–488. 2105276 10.1128/iai.58.2.480-488.1990 PMC258482

[24] Florin I, Thelestam M (1986) Lysosomal involvement in cellular intoxication with Clostridium difficile toxin B. Microb Pathog 1: 373–385. 3508493 10.1016/0882-4010(86)90069-0

[25] Papatheodorou P, Zamboglou C, Genisyuerek S, Guttenberg G, Aktories K (2010) Clostridial glucosylating toxins enter cells via clathrin-mediated endocytosis. PLoS One 5: e10673. 20498856 10.1371/journal.pone.0010673 PMC2871790

[26] Giesemann T, Jank T, Gerhard R, Maier E, Just I, et al (2006) Cholesterol-dependent pore formation of Clostridium difficile toxin A. J Biol Chem 281: 10808–10815. 16513641 10.1074/jbc.M512720200

[27] Barth H, Pfeifer G, Hofmann F, Maier E, Benz R, et al (2001) Low pH-induced formation of ion channels by clostridium difficile toxin B in target cells. J Biol Chem 276: 10670–10676. 11152463 10.1074/jbc.M009445200

[28] Qa'Dan M, Spyres LM, Ballard JD (2000) pH-induced conformational changes in Clostridium difficile toxin B. Infect Immun 68: 2470–2474. 10768933 10.1128/iai.68.5.2470-2474.2000 PMC97448

[29] Reineke J, Tenzer S, Rupnik M, Koschinski A, Hasselmayer O, et al (2007) Autocatalytic cleavage of Clostridium difficile toxin B. Nature 446: 415–419. 17334356 10.1038/nature05622

[30] Pfeifer G, Schirmer J, Leemhuis J, Busch C, Meyer DK, et al (2003) Cellular uptake of Clostridium difficile toxin B. Translocation of the N-terminal catalytic domain into the cytosol of eukaryotic cells. J Biol Chem 278: 44535–44541. 12941936 10.1074/jbc.M307540200

[31] Geissler B, Tungekar R, Satchell KJ (2010) Identification of a conserved membrane localization domain within numerous large bacterial protein toxins. Proc Natl Acad Sci U S A 107: 5581–5586. 20212166 10.1073/pnas.0908700107 PMC2851783

[32] Egerer M, Giesemann T, Jank T, Satchell KJ, Aktories K (2007) Auto-catalytic cleavage of Clostridium difficile toxins A and B depends on cysteine protease activity. J Biol Chem 282: 25314–25321. 17591770 10.1074/jbc.M703062200

[33] Pruitt RN, Chagot B, Cover M, Chazin WJ, Spiller B, et al (2009) Structure-function analysis of inositol hexakisphosphate-induced autoprocessing in Clostridium difficile toxin A. J Biol Chem 284: 21934–21940. 19553670 10.1074/jbc.M109.018929 PMC2755918

[34] Puri AW, Lupardus PJ, Deu E, Albrow VE, Garcia KC, et al (2010) Rational design of inhibitors and activity-based probes targeting Clostridium difficile virulence factor TcdB. Chem Biol 17: 1201–1211. 21095570 10.1016/j.chembiol.2010.09.011 PMC3005307

[35] Shen A, Lupardus PJ, Gersch MM, Puri AW, Albrow VE, et al (2011) Defining an allosteric circuit in the cysteine protease domain of Clostridium difficile toxins. Nat Struct Mol Biol 18: 364–371. 21317893 10.1038/nsmb.1990 PMC3076311

[36] Savidge TC, Urvil P, Oezguen N, Ali K, Choudhury A, et al (2011) Host S-nitrosylation inhibits clostridial small molecule-activated glucosylating toxins. Nat Med 17: 1136–1141. 21857653 10.1038/nm.2405 PMC3277400

[37] Lanis JM, Hightower LD, Shen A, Ballard JD (2012) TcdB from hypervirulent Clostridium difficile exhibits increased efficiency of autoprocessing. Mol Microbiol 84: 66–76. 22372854 10.1111/j.1365-2958.2012.08009.x PMC3313004

[38] Jank T, Giesemann T, Aktories K (2007) Clostridium difficile glucosyltransferase toxin B-essential amino acids for substrate binding. J Biol Chem 282: 35222–35231. 17901056 10.1074/jbc.M703138200

[39] Kreimeyer I, Euler F, Marckscheffel A, Tatge H, Pich A, et al (2011) Autoproteolytic cleavage mediates cytotoxicity of Clostridium difficile toxin A. Naunyn Schmiedebergs Arch Pharmacol 383: 253–262. 21046073 10.1007/s00210-010-0574-x

[40] Ryder AB, Huang Y, Li H, Zheng M, Wang X, et al (2010) Assessment of Clostridium difficile infections by quantitative detection of tcdB toxin by use of a real-time cell analysis system. J Clin Microbiol 48: 4129–4134. 20720023 10.1128/JCM.01104-10 PMC3020809

[41] Hewlett EL, Donato GM, Gray MC (2006) Macrophage cytotoxicity produced by adenylate cyclase toxin from Bordetella pertussis: more than just making cyclic AMP!. Mol Microbiol 59: 447–459. 16390441 10.1111/j.1365-2958.2005.04958.x

[42] Pruitt RN, Chambers MG, Ng KK, Ohi MD, Lacy DB (2010) Structural organization of the functional domains of Clostridium difficile toxins A and B. Proc Natl Acad Sci U S A 107: 13467–13472. 20624955 10.1073/pnas.1002199107 PMC2922184

[43] Harlow EaL, D (1988) Antibodies: a laboratory manual. Cold Spring Harbor, NY: Cold Spring Harbor.

[44] Riegler M, Sedivy R, Pothoulakis C, Hamilton G, Zacherl J, et al (1995) Clostridium difficile toxin B is more potent than toxin A in damaging human colonic epithelium in vitro. J Clin Invest 95: 2004–2011. 7738167 10.1172/JCI117885 PMC295778

