# Correction: *Clostridium difficile* Toxin B Causes Epithelial Cell Necrosis through an Autoprocessing-Independent Mechanism

**DOI:** 10.1371/annotation/f9017013-88c8-44db-818b-08b9322f3814

**Published:** 2012-12-18

**Authors:** Nicole M. Chumbler, Melissa A. Farrow, Lynne A. Lapierre, Jeffrey L. Franklin, David B. Haslam, James R. Goldenring, D. Borden Lacy

The fifth author's name was missing the middle initial. The correct name is: David B. Haslam. The correct citation is: Chumbler NM, Farrow MA, Lapierre LA, Franklin JL, Haslam DB, et al. (2012) Clostridium difficile Toxin B Causes Epithelial Cell Necrosis through an Autoprocessing-Independent Mechanism. PLoS Pathog 8(12): e1003072. doi:10.1371/journal.ppat.1003072

