# Supplementary figures and images for: Clostridium difficile Toxin B Causes Epithelial Cell Necrosis through an Autoprocessing-Independent Mechanism
Source: PLoS Pathog. 2012 Dec 6;8(12):e1003072. doi: 10.1371/journal.ppat.1003072 (PMC3516567; doi:10.1371/journal.ppat.1003072)

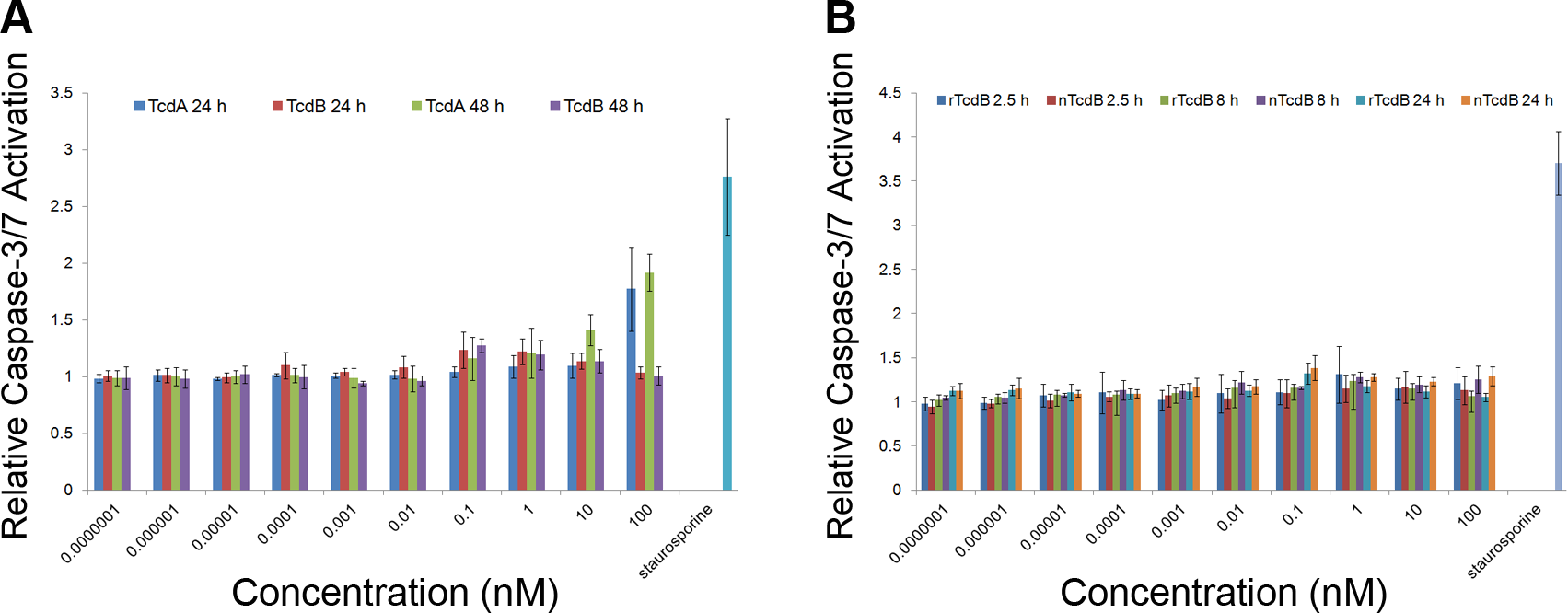

Supplement: Figure S1 — TcdA activates caspase-3/7 while both recombinant and native TcdB do not. A, TcdB does not induce caspase-3/7 activation in HeLa cells, as detected by a fluorescent indicator, Apo-One, at 24 or 48 h. TcdA, however, does induce caspase-3/7 activation at a concentration of 100 nM at 24 h and 10 and 100 nM at 48 h. B, TcdB purified from C. difficile supernatant looks similar to TcdB purified from B. megaterium in that neither induce caspase-3/7 activation. Values represent the average of 3 independent experiments in which each condition was tested in triplicate. Error bars represent the standard deviation of the average of the three independent experiments. (TIF) [file ppat.1003072.s001.tif]

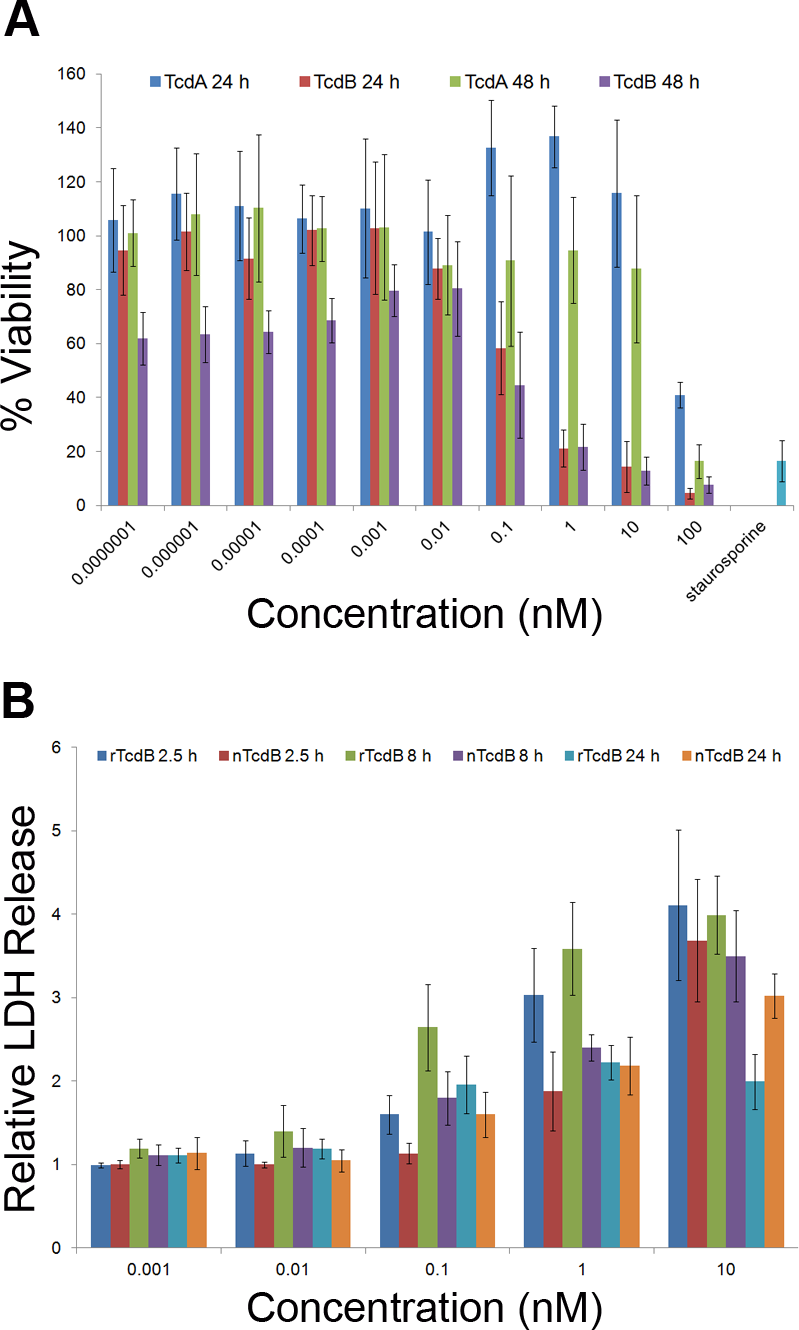

Supplement: Figure S2 — TcdB is more cytotoxic than TcdA, and the effects of native and recombinant TcdB on LDH release are similar. A, TcdB induces significant HeLa cell death, as detected by CellTiterGlo, in 24 h at concentrations of 1, 10, and 100 nM. At 48 h, a loss of cell viability was observed at lower concentrations in a dose-independent fashion. TcdA induces significant cell death at 24 h and 48 h at a concentration of 100 nM. B, TcdB purified from C. difficile and B. megaterium induce release of LDH starting at 2.5 h, with increased levels apparent after 8 h of treatment. Values represent the average of 3 independent experiments in which each condition was tested in triplicate. Error bars represent the standard deviation of the three independent experiments. (TIF) [file ppat.1003072.s002.tif]

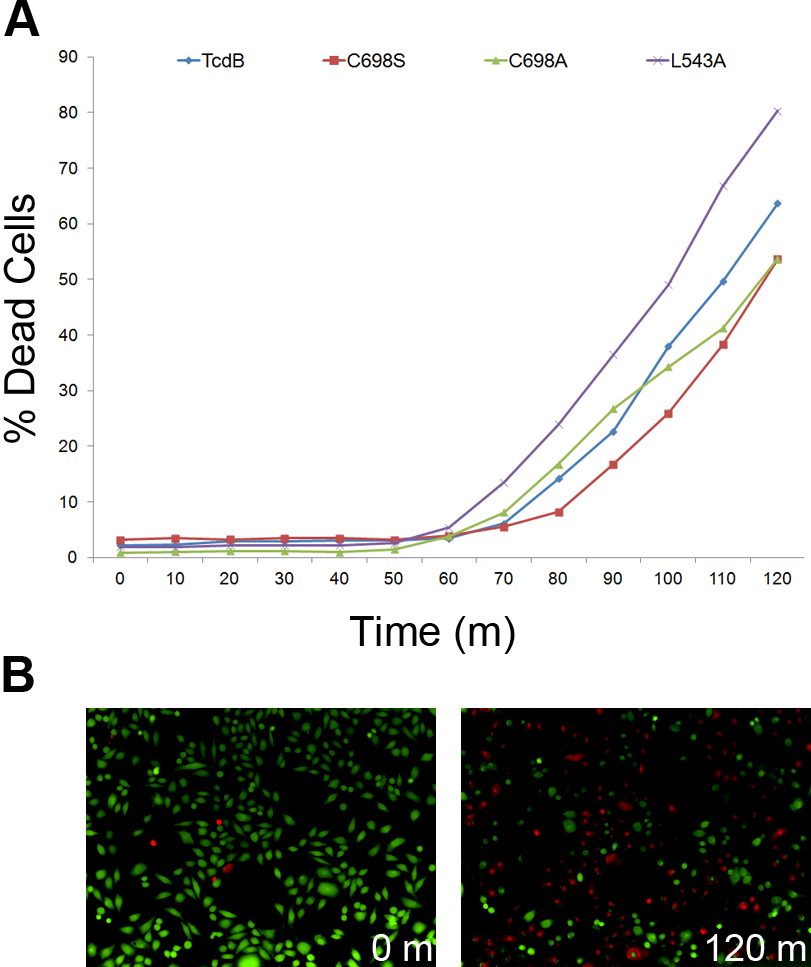

Supplement: Figure S3 — TcdB and TcdB autoprocessing mutants have the same cytotoxicity kinetics. A, TcdB, TcdB C698S, TcdB C698A, and TcdB L543A at 10 nM induce HeLa cell death at similar rates, as detected by Live/Dead Cell Imaging dyes. Values represent the number of red (dead) cells per total number of cells (red+green) over six image fields and were calculated using Columbus Analysis Software. Dead cells were defined as having a red intensity greater than 450 relative units. B, Representative pictures of TcdB treated cells at 0 and 120 minutes. Images were taken using an Opera High-Throughput Confocal Screening Microscope. (TIF) [file ppat.1003072.s003.tif]

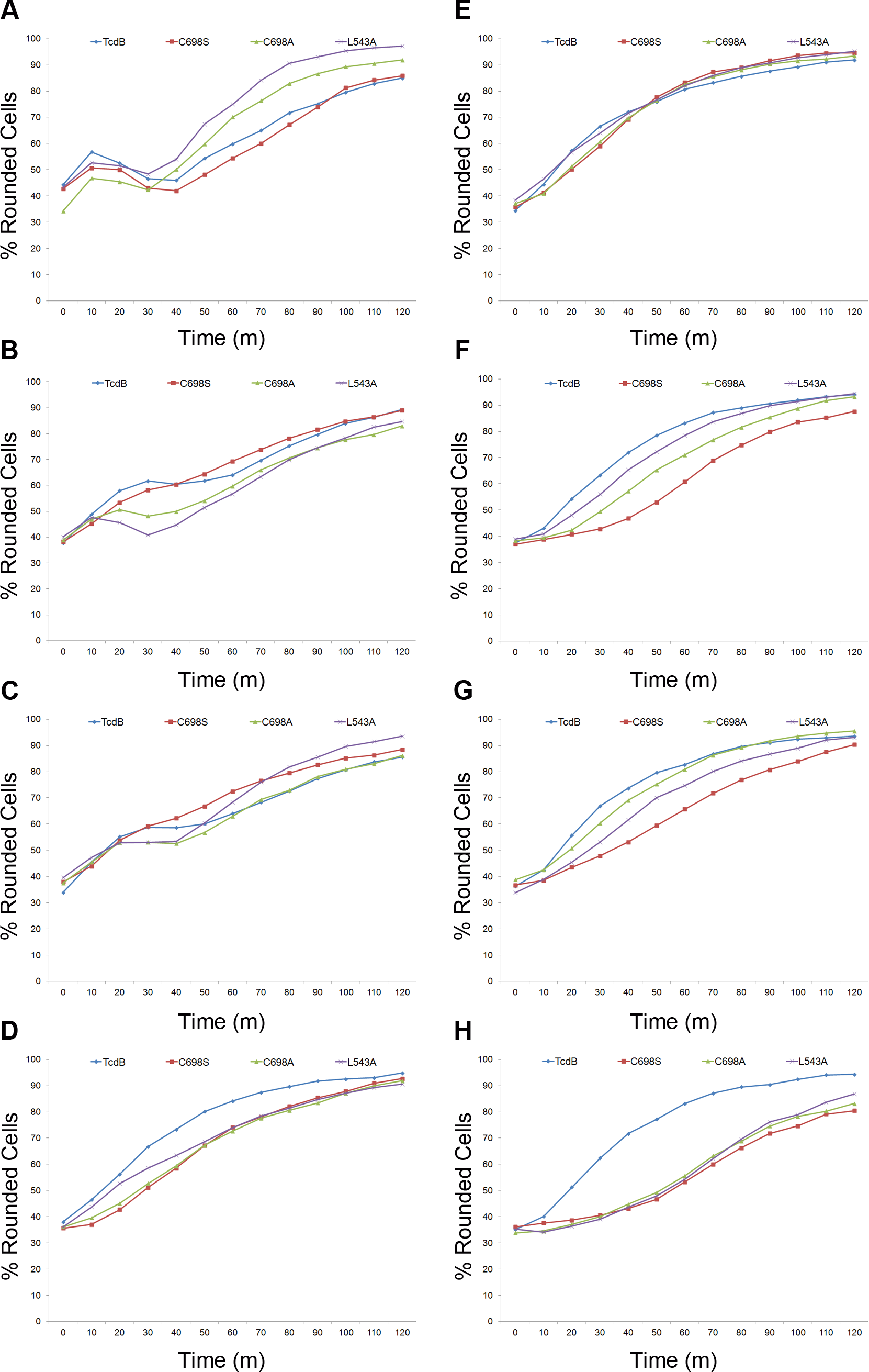

Supplement: Figure S4 — TcdB and TcdB autoprocessing mutants have different cytopathic kinetics at 1 fM. HeLa cells were treated with multiple concentrations of wild-type and mutant TcdB proteins and imaged every 10 minutes over a 2 hour time course. The percentage of round cells was quantified over six fields for each concentration and time point. Percent rounded cells induced by TcdB and autoprocessing mutants is shown at concentrations of A, 10 nM, B, 1 nM, C, 100 pM, D, 10 pM, E, 1 pM, F, 100 fM, G, 10 fM, and H, 1 fM. Differences in the rounding kinetics between TcdB and autoprocessing mutants begin to appear at a concentration of 100 fM and are clearly distinct at 1 fM. Images were collected with an Opera High-Throughput Confocal Screening Microscope in an environment-controlled chamber at 37°C, 5% CO2. Round cells were defined as having an area less than 500 um2 and a width-to-length ratio greater than 0.4. Analysis was performed using Columbus Analysis software. (TIF) [file ppat.1003072.s004.tif]
